# One-year dataset of hourly air quality parameters from 100 air purifiers used in China residential buildings

**DOI:** 10.1038/s41597-023-02640-y

**Published:** 2023-10-18

**Authors:** Jiaze Wei, Yan Wang, Jinhan Mo, Cheng Fan

**Affiliations:** 1 Beijing Key Laboratory of Indoor Air Quality Evaluation and Control, Department of Building Science, Tsinghua University, Beijing, 100084 China; 2 College of Civil and Transportation Engineering, Shenzhen University, Shenzhen, 518060 China; 3 Key Laboratory of Coastal Urban Resilient Infrastructures (Shenzhen University), Ministry of Education, Shenzhen, 518060 China; 4 Key Laboratory of Eco Planning & Green Building (Tsinghua University), Ministry of Education, Beijing, 100084 China

**Keywords:** Environmental monitoring, Pollution remediation

## Abstract

Household air purifiers have been widely used as an effective approach to improving indoor air quality. Air purifiers can automatically record indoor air quality parameters, providing valuable data resources for in-depth data-driven analysis. This work presents a one-year hourly indoor air quality dataset collected by household air purifiers in 100 residential homes in 18 provinces across 4 different climate zones in China. The data were collected from July 1, 2021, to July 1, 2022. The concentrations of formaldehyde, PM_2.5_, TVOC, temperature, relative humidity, on/off status and the airflow rate of air purifiers during operations were recorded hourly. The data were carefully screened with possibly missing values imputed using chained equation-based methods if any. The dataset provides a comprehensive and detailed picture of the indoor air quality in residential buildings, enabling evaluations on the cleaning effect of air purifiers, the impact of outdoor climate change on indoor air quality, and the future trends in indoor human behavior.

## Background & Summary

Indoor air pollution has become a major global public health threat that requires increasing joint efforts from policymaking, academic research, industrial innovations, etc. In most cases, indoor air quality is more polluted than the atmospheric environment^1^. For instance, the concentrations of particulate matter (PM), formaldehyde (HCHO), and volatile organic compounds (VOCs) in indoor air can occasionally be several times higher than those found in outdoor air, imposing substantial health threats to indoor occupants^2,3^. Formaldehyde and PM are categorized as carcinogenic substances and could cause severe diseases such as lung cancer, given long-term exposures^4^. Existing research indicated that such exposure to indoor air pollution could contribute to excess deaths in developing countries, accounting for 4% of the global disease burden^5^. Other studies also validated the adverse effects of VOCs and particle exposure on public health, such as chronic body damages^6–8^, cancer^9–11^, heart diseases^12–14^, lung diseases^15,16^, and skin inflammations^17^.

Household air purifiers can improve indoor air quality by reducing concentrations of harmful pollutants in indoor air, such as fine particles, harmful gases and bacteria^18^. The application of air purifiers is therefore becoming increasingly popular in residential buildings. For instance, in China, a study revealed that the use of air purifiers could significantly reduce indoor PM_2.5_ concentrations and positively affect population health^19^. Zhang *et al*.^20^ reviewed relevant research on applying air filtration technologies to improve indoor air quality, which used mechanical filters to efficiently remove larger particles, and adopted electrostatic force-enhanced filtration to achieve large dust loading capacity and ultralow pressure drops^21^. Tian *et al*.^22^ provided a comprehensive summary of the working principles and performance of novel electrostatic force-enhanced filtration technologies. In their analysis, Tian *et al*. emphasized the importance of considering both the initial and long-term performance of electrostatic force-enhanced filtration technologies. They found that charging PM or filters could significantly improve filtration efficiency and overall performance. Furthermore, the performance of these technologies was found to vary when exposed to non-oily particles, oily particles, and bioaerosols. As alternatives, adsorption technologies have also gained increasing popularity in air purifiers due to their efficient and affordable natures^23,24^, although the pollutant removal capability may vary given various sorbents. Chen *et al*.^25^ thoroughly reviewed the methods for enhancing mass transfer in gas phase adsorption catalysis using the Direct Ink Writing (DIW) technique. The review encompassed various aspects such as raw materials, preparation processes, auxiliary optimization methods, and practical applications. In addition, the review also discussed the potential prospects and challenges of employing DIW methods to achieve efficient mass transfer kinetics. Air purifier efficiency has been greatly improved along with these filtration and adsorption technology improvements. As a result, air purifiers have been widely used to reduce indoor pollutant concentrations, improve indoor air quality and reduce the risk of associated diseases.

Air purifiers are typically equipped with various sensors for real-time measurements of formaldehyde, carbon dioxide, VOCs, etc. Currently, most studies focus on evaluating the environmental changes before and after using air purifiers while using external sensors rather than built-in sensors in air purifiers. Huang *et al*.^26^ used indoor PM concentration changes to develop predictive models for automated and real-time controls over air purifiers. Cooper *et al*.^27^ utilized the relationship between air purifier operations and indoor PM concentrations to make inferences on indoor occupant behavior and guide the use of air purifiers. Using indoor environmental parameters collected, Pei *et al*.^28^ analyzed the operational behavior of portable air purifiers in Chinese residences and evaluated their overall performance in improving indoor air quality. Malayeri *et al*.^29^ utilized neural networks and genetic algorithms to design photocatalytic reactors for air purifiers. Previous studies have shown that more detailed data on air purifiers are helpful for developing improved predictive models, facilitating product design, and guiding human behavior inferences.

Utilizing real-time data collected by built-in sensors in air purifiers, prediction models on indoor pollutant concentrations can be readily established to facilitate optimal designs and operation of air purifiers. Moreover, leveraging experiences learned in diverse environments, artificial intelligence models can be developed for the easy and quick adaptation of air purifiers in new settings. At present, there is a scarcity of datasets to provide comprehensive coverages and descriptions of indoor environmental parameters and the corresponding air purifier operating conditions. This paper presents a dataset related to air purifier operations as a solution. The uniqueness of the dataset lies in the collection of one-year hourly data from 100 air purifiers in residential buildings dispersed across 18 provinces in China. The dataset includes the air purifier’s working status and airflow rate together with 5 monitoring variables, i.e., formaldehyde concentration, PM_2.5_ concentration, total volatile organic compound (TVOC) concentration, temperature, and relative humidity recorded. Such dataset can be used for the following applications:

- To derive optimal design guidance for new air purifiers.
- To guide personnel use by indoor human behavior predictions.
- To develop and validate prediction models on indoor pollutant emissions.
- To perform area-level data analysis using statistical or machine learning algorithms.
- To develop cost-effective control methods for air purifier operations.
- To detect and diagnose faults in air purifier operations.

## Methods

### Data acquisition and anonymization

**Fig. 1:**
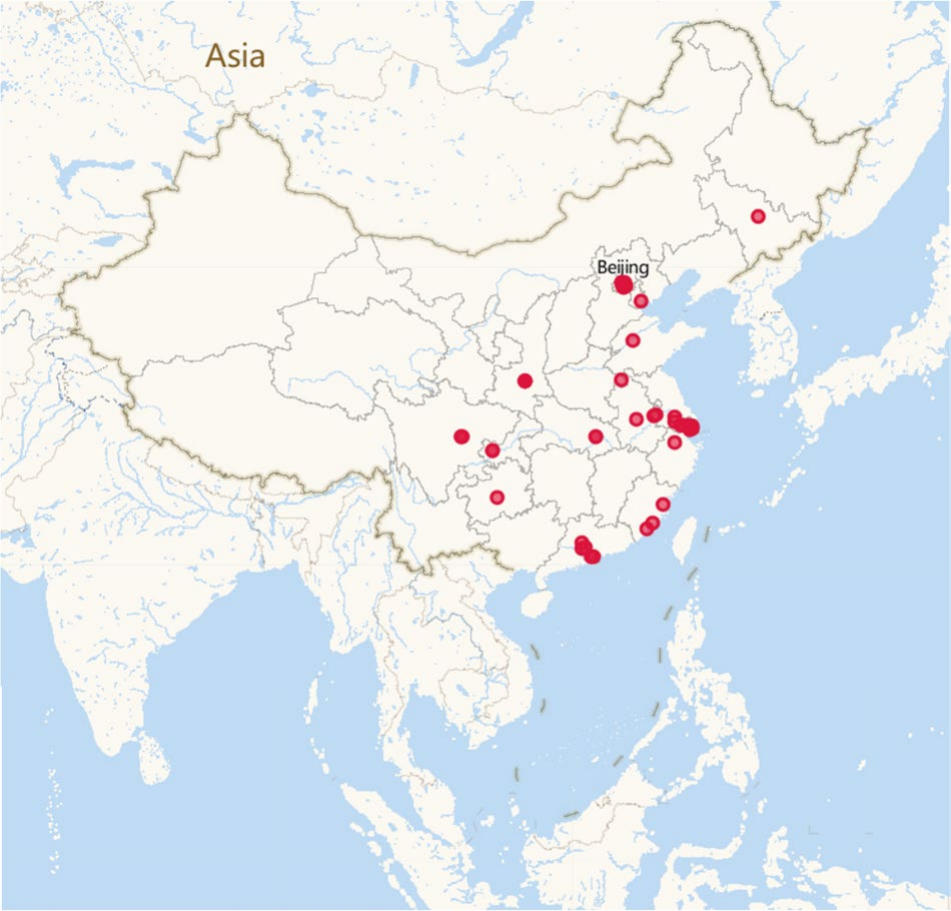
Air purifiers in 18 provinces across 4 different climate zones in China. Each translucent red circle represents an air purifier; the darker the circle colour, the more intensively the area is used.

The original dataset was collected from July 1, 2021, to July 1, 2022, and was sourced from hourly data collected by 100 air purifiers situated across 18 Chinese provinces (Fig. 1). Detailed information on the distribution of air purifiers can be found in Table 1. Beijing 352 Environmental Protection Technology Co. LTD provided the raw information to the authors via collaboration. The specified air purifier model is the X86 Smart Air Purifier. Interested researchers can access the raw data and possessed dataset for free by visiting the website in the “Data Records” section. The raw data were collected by various built-in sensors installed inside the air purifier. Before using the purifier, residents were asked if they agreed on the data collection, sharing, and publishing of the measurements collected in their homes. After obtaining the user’s consent, the anonymized air purifier will record the current indoor air parameters every hour during operations and upload them to the cloud for data storage via Wi-Fi. The collected data have been anonymized to avoid possible violations of privacy.

**Table 1:** Air purifier distribution details.

Cities	Climate Zones	Number of air purifiers
Jilin	Severe Cold	1
Beijing	Cold	13
Chongqing	Cold	2
Jinan	Cold	1
Shangqiu	Cold	1
Tianjin	Cold	1
Xi’an	Cold	5
Chengdu	Hot Summer&Cold Winter	3
Fuzhou	Hot Summer&Cold Winter	1
Hangzhou	Hot Summer&Cold Winter	1
Hefei	Hot Summer&Cold Winter	1
Nanjing	Hot Summer&Cold Winter	2
Shanghai	Hot Summer&Cold Winter	10
Suzhou	Hot Summer&Cold Winter	3
Wuhan	Hot Summer&Cold Winter	2
Wuxi	Hot Summer&Cold Winter	2
Guiyang	Temperate	1
Guangzhou	Hot Summer&Warm Winter	3
Quanzhou	Hot Summer&Warm Winter	1
Shenzhen	Hot Summer&Warm Winter	3
Xiamen	Hot Summer&Warm Winter	1
Not mentioned		42
Total		100

As summarized in Table 2, the dataset consists of nine variables in total. The first three variables represent the data collection timestamps, machine ID and on/off status. The remaining six variables represent measurements of airflow rate and indoor air quality. The original 5582 datasets provided by the company were used to screen 100 air purifiers, with a maximum missing data ratio of 7%. The data were recorded in 100 CSV files, each containing around 8760 rows. The project team has contacted potential data providers with the following requirements to ensure validity in the preliminary data collection phase:

- The data must be completely derived from the designated site to reflect actual occupant behaviors in real buildings.
- The dataset should contain all five air quality parameters (formaldehyde concentration, PM_2.5_ concentration, TVOC concentration, temperature, and relative humidity), and the data should be traceable to its corresponding machine.
- The total length of the hourly data should be no less than one year.

**Table 2:** Columns of the raw and processed data, including their reporting resolution.

Original column name	English name	Description	Resolution
date_time	Data time	Recording time of data	1 h
iot_id	Machine id	Machine-exclusive serial number	—
power_switch	On/off status	On/off status at this moment	—
wind_value	Airflow rate	Airflow rate at this moment	1 m^3^/h
pm25	PM_2.5_ concentration	Average PM_2.5_ concentration in the past hour	1 μg/m^3^
hcho	Formaldehyde concentration	Average Formaldehyde concentration in the past hour	1 μg/m^3^
tvoc	TVOC concentration	The average TVOC concentration in the past hour	1 μg/m^3^
temperature	Temperature	The average temperature in the past hour	1 °C
humidity	Relative humidity	Average relative humidity concentration in the past hour	1 %

### Data selection and processing

**Fig. 2:**
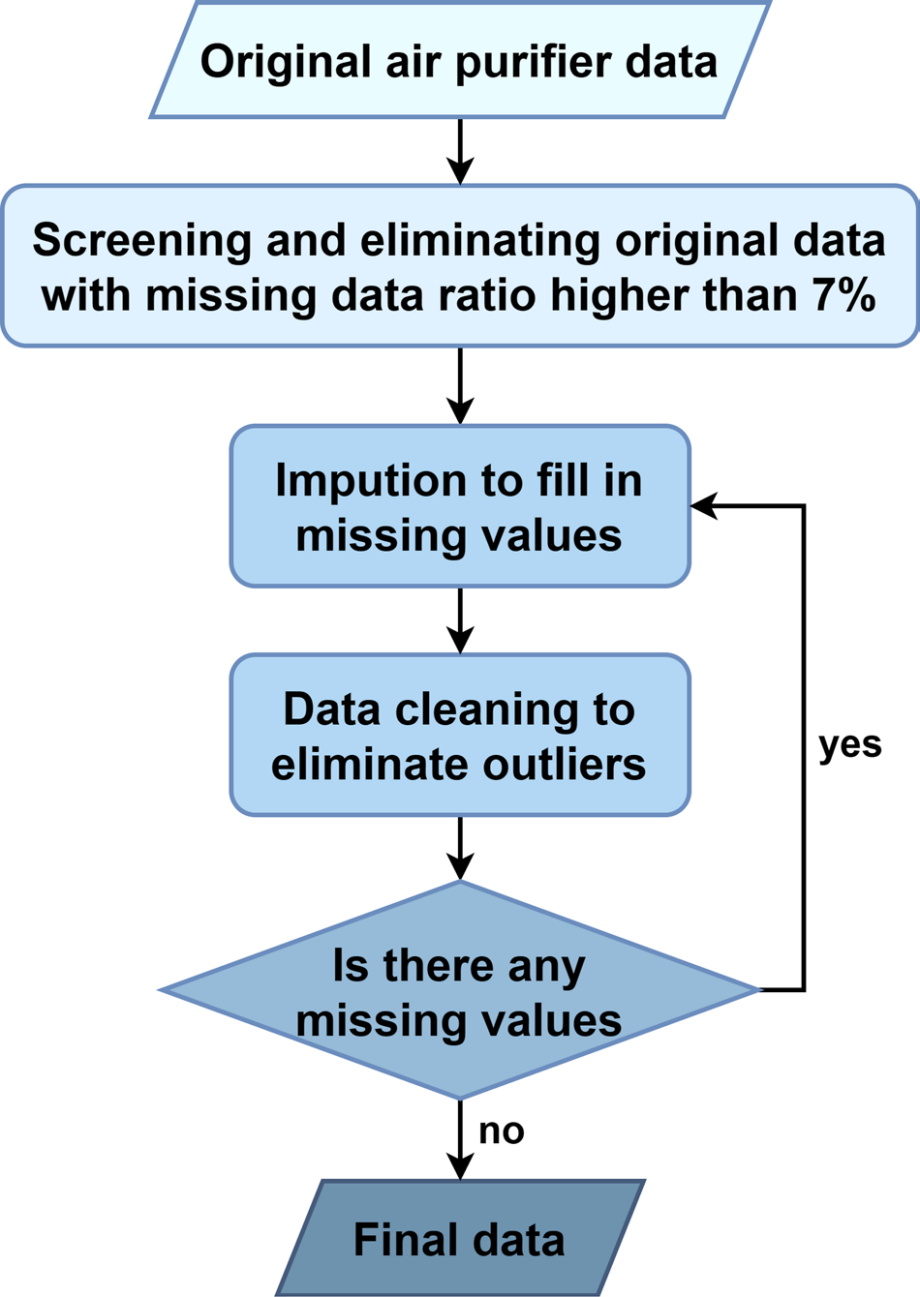
Data processing flowchart.

The processing of the original data is shown in Fig. 2. Generally, a missing rate below 10% is deemed as low. In order to ensure improved data stability through subsequent data interpolation, a threshold of 7% is selected for the initial data screening. To ascertain the missing rate for each parameter in each air purifier, we evaluate the absence of each distinct parameter and identify the highest missing rate as the predominant absence rate for that specific air purifier. Subsequently, any missing rates exceeding 7% are excluded from the collective data pool and retained as part of the initial dataset. After the initial data is established, the missing values are filled in. Afterward, data cleaning methods were implemented to remove and impute data outliers. This iterative process is repeated until the dataset contains no missing values.

## Data Records

The dataset is available on figshare^30^ [10.6084/m9.figshare.24278101]. The raw data is organized in a zipped file named “anonymized_raw_data”. The file size is 272MB (3.90GB before compression). It contains 5582 csv files with anonymized raw data, the primary data obtained from Beijing 352 Environmental Protection Technology Co. LTD. The zipped file named “processed_final_data” contains 100 csv files, each including the data using the processing method mentioned above. The above two zip files are packaged in a file called “One-year dataset of hourly air quality parameters from 100 air purifiers used in China residential buildings”. All the files in both folders cover the data collection period from July 1, 2021, to July 1, 2022. The only difference among the machines lies in their usage locations, with the model number and duration of use remaining consistent across all of them. The csv file named “Machine parameters (with ID and Cities)” contains the iot_id and its corresponding city information about each air purifier. The data are also available at github [https://github.com/weijiaze/Scientific-data].

## Technical Validation

### Data pre-processing

#### Data selection

**Fig. 3:**
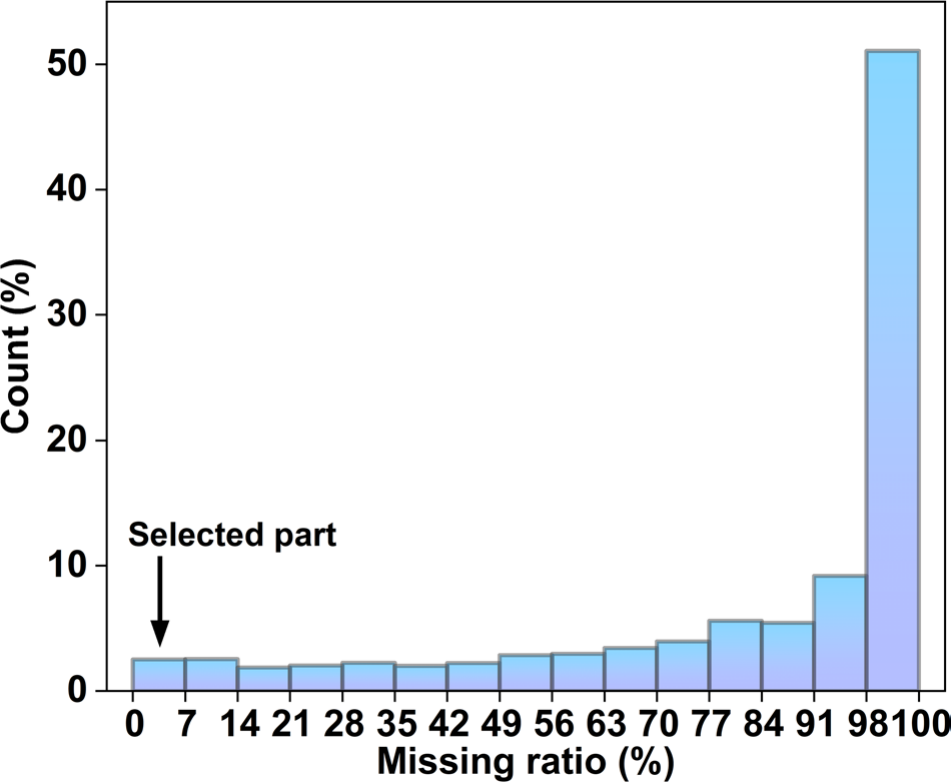
Distribution of missing ratio for raw data, The x-axis indicates the missing ratio missing values, the interval is 7, the y-axis indicates the number of air purifiers contained in the interval, and the final selection is one hundred air purifiers in 0–7% as the initial data.

As shown in Fig. 3, most of the raw datasets exhibit substantial missing values. To ensure the reliability of data imputation, this study specifically chooses raw datasets with less than 7% missing values for analysis, as mentioned in Fig. 2.

#### Data imputation

In this study, missing value imputation methods have been applied to two groups of data separately, i.e., one for on/off status, airflow rate and the other for air quality parameters.

##### a. imputation of on/off status and airflow rate

Based on data exploration, it is found that the on/off status and airflow rate will become missing at the same time due to random discontinuous data loss. Therefore, the following method has been utilized to induce missing data. An example will illustrate the imputation of missing data in the on/off state as follows, and the airflow rate will be imputed using the same approach. (1) If there is no change in the on/off status (i.e., denoted as 1 or 0) before and after the missing data, the missing data is considered to be consistent with adjacent measurements. In this case, the Next Observation Carried Backward (NOCB) method is used for data imputation. It uses the closest data backward to fill in the missing data. It involves filling in the current missing value using one of its preceding data points. (2) If the on/off status before and after the missing data varies, the on/off status of the missing data will be inferred based on indoor air quality measurements. As an example, assuming the on/off status of the previous non-missing data sample is off (i.e., denoted as 0) and the measurements on indoor air quality (e.g., formaldehyde concentration) are reducing, the on-off status will be imputed as on (i.e., denoted as 1). The same method can be applied to the versa situations.

##### b. imputation of air quality parameters

Multivariate imputation techniques can offer significantly improved capabilities in filling substantial gaps within the dataset by transforming time series data into matrices, with each row representing a weekly cycle. Although MICE necessitates substantially greater computational resources, it demonstrates exceptional robustness. Irrespective of sensor categories, missing rates, or gap sizes, MICE consistently produces imputations with an average NRMSE of 1 standard deviation for each sensor^31^. In terms of concentrations of formaldehyde, PM_2.5_, TVOC, temperature, and relative humidity, the Multiple Imputation by Chained Equations (MICE)^32,33^ has been used for data imputation. Prediction models have been developed to estimate the missing data using other column data near the missing data as features. More specifically, linear regression is employed for predicting continuous missing values, while logistic regression is utilized for classifying categorical missing values.

#### Data cleaning

The PauTa criterion^34^, a statistical method to identify outliers beyond triple standard deviations, is used for outlier detection in this study. The PauTa criterion was dynamically adjusted based on a sliding window of 7 days. Any outlier identified using this criterion will be replaced using the above data imputation methods.

When the data originate from a single non-randomized case study, measurement accuracy is critical because no alternate use cases may be utilized to identify outliers and filler approaches. The validity of the missing data filler is more demanding in the data used because the missing data are more scattered. This research utilizes the MICE interpolation method to accurately estimate missing data points by considering the distribution of the original dataset. This method generates a series of complete datasets, typically ranging from 3 to 10, by filling in the missing values through interpolation techniques based on the values present in the original dataset. Subsequently, standard statistical methods are applied to each complete dataset, and the outcomes of these separate analyses are combined to form a comprehensive set of results. Finally, the interpolation results are compared to the original distribution curve, and the interpolation result that best fits the curve is selected as the final data point. This study generates 10 interpolation results and selects the one that best fits the original distribution curve according to the fit of the interpolation results as the final data. After the comparison of imputation results and raw data in distribution, it seems that the data distribution after interpolation is the same as before, which greatly improves the reliability of the data set. Overall, the MICE interpolation method provides a robust approach for handling missing data by generating multiple complete datasets and utilizing statistical techniques to integrate the results. It ensures a more accurate and comprehensive estimation of missing values, enhancing the validity and reliability of subsequent analyses.

**Fig. 4:**
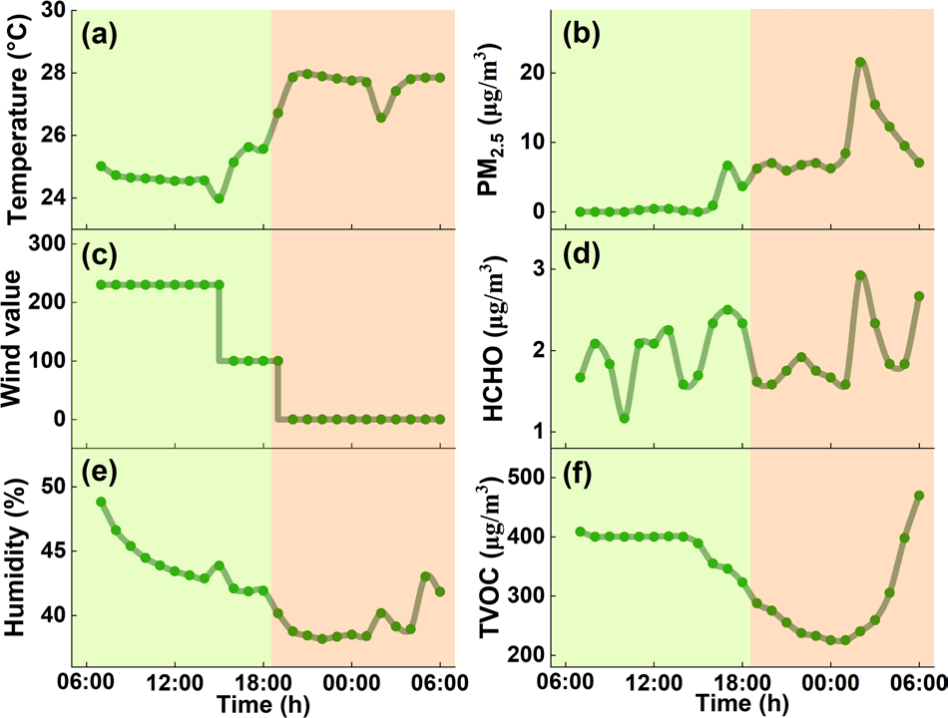
Change in all monitored parameters of the air purifier in one day (November 25, 2021, 7:00 am to November 26, 2021, 6:00 am). The green shadow means the air purifier is on during the period, and the orange shadow means the air purifier is off.

To accurately present processed data, it is essential to eliminate outliers and missing values. The resulting graph will display gradual and consistent curves without sudden or steep fluctuations by removing these outliers and missing values. This step is crucial in accurately representing the processed data and clearly depicting the trends. Figure 4 shows the variations of all processed parameters of a specific air purifier installed in Shaanxi Province in one day. It indicates that the temperature and humidity fluctuate regularly with time, while the peak of air pollutants usually appears at around 9:00 am and 9:00 pm, and the air purifier gears are also adjusted accordingly to changes in pollutant concentrations. Around noon, the pollutant concentration typically decreases to a lower value, leading to the automatic shutdown of the purifier. That is why the peak pollutant concentration at night is higher than that during the day.

**Fig. 5:**
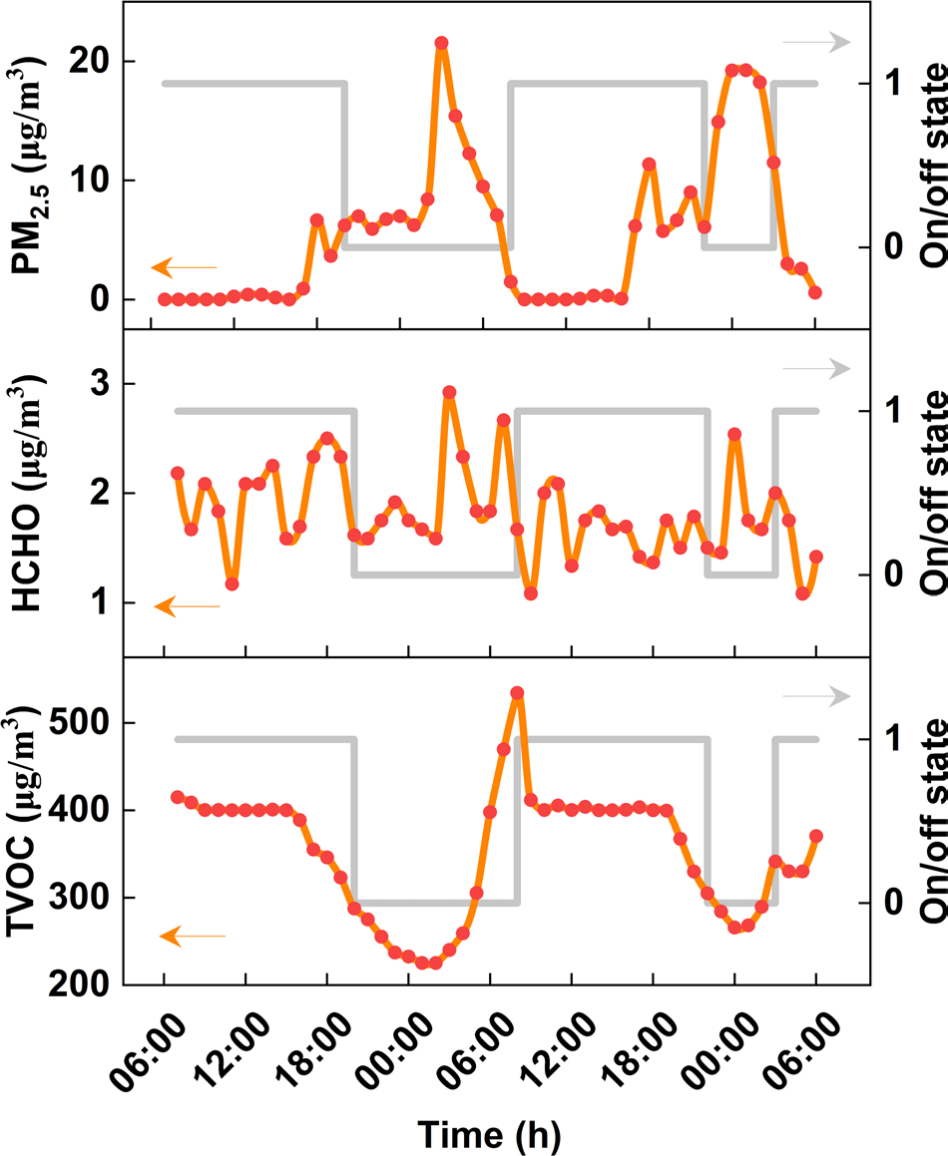
Indoor air quality parameters (PM_2.5_, HCHO, TVOC) coupled with the on/off status in two days (November 25, 2021, 6:00 am to November 27, 2021, 5:00 am), When on/off status = 1, the air purifier is on; when on/off status = 0, the air purifier is off.

Figure 5 shows another example of parameter variations with the on/off status over a 2-day period from the same device installed in Shaanxi Province. The sampling time was set to one hour, and the air purifier was set to auto-regulation mode, recording the average concentration of pollutants within each hour and the corresponding status of the air purifier. It can be seen that the air purifier will automatically turn on when the pollutant concentration rises above the threshold until it reaches the normal concentration, which presents rather good control ability on indoor pollutants.

## Usage Notes

Based on the currently available dataset, which can serve as a foundation for future investigations, we can utilize variations in indoor pollutant concentrations to discern specific occupant behaviors within the enclosed space and ascertain the occupant’s daily schedule^35^. For instance, if there is a sudden surge in pollutant concentration at 12:00 noon or 6:00 pm, coinciding with the activation of an air purifier, we can infer that this spike is likely attributed to cooking activities by the user. Conversely, if the air purifier remains inactive while pollutant levels decrease concurrently with shifts in temperature and humidity, it could be hypothesized that the occupant has opened doors or windows. Furthermore, we can retrieve real-time local data on temperature, humidity, and pollutant concentration and compare it against the data from the air purifier itself. This comparative analysis allows us to validate the air purifier’s efficacy in improving indoor air quality over time.

## Data Availability

Each CSV file has a large amount of data. Programming languages (such as Python) for data visualization and manipulation are recommended for data exploration and analysis. The data preprocessing codes are written in Python on Jupyter Notebook, which can be found at “https://github.com/weijiaze/Scientific-data/blob/master/imputefile.ipynb. The code runs on a Windows computer using Python 3. The Python 3 codes to reproduce the examples provided in this article can be found at https://github.com/weijiaze/Scientific-data.

## References

[1] Carslaw N (2007). A new detailed chemical model for indoor air pollution. Atmos. Environ..

[2] National Institute for Health and Care Excellence. *Indoor air quality at home*, https://www.nice.org.uk/guidance/ng149 (2020).

[3] United States Environmental Protection Agency. *Indoor Air Quality: What are the trends in indoor air quality and their effects on human health?* https://www.epa.gov/report-environment/indoor-air-quality (2017).

[4] Herbarth O, Schlink U, Muller A, Richter M (2003). Spatiotemporal distribution of airborne mould spores in apartments. Mycol. Res..

[5] Bruce N, Perez-Padilla R, Albalak R (2000). Indoor air pollution in developing countries: a major environmental and public health challenge. B. World Health Organ..

[6] Lei, X., Chen, Q. W., Wang, Y. & Mo, J. H. Modelling and implementation of an *in-situ* thermally regenerated adsorption module for removing gaseous xylene. *Build. Environ*. (2023).

[7] Chen QW, Tian EZ, Luo ZY, Mo JH (2022). Adsorption film with sub-milli-interface morphologies via direct ink writing for indoor formaldehyde removal. J. Hazard. Mater..

[8] Wang, Y., Yu, T. & Mo, J. H. The influence of indoor environmental factors on toluene uptake rate of a tube-type diffusive sampler. *J. Build. Eng*. **54** (2022).

[9] Cheek E, Guercio V, Shrubsole C, Dimitroulopoulou S (2021). Portable air purification: Review of impacts on indoor air quality and health. Sci. Total. Environ..

[10] Liang HY (2023). Association of outdoor air pollution, lifestyle, genetic factors with the risk of lung cancer: A prospective cohort study. Environ. Res..

[11] Urman A, Hosgood HD (2015). Lung cancer risk, genetic variation, and air pollution. Ebiomedicine.

[12] Yang BY (2018). Global association between ambient air pollution and blood pressure: A systematic review and meta-analysis. Environ. Pollut..

[13] Liu S (2018). Cardiovascular benefits of short-term indoor air filtration intervention in elderly living in Beijing: An extended analysis of BIAPSY study. Environ. Res..

[14] Xia X (2021). Effectiveness of indoor air purification intervention in improving cardiovascular health: A systematic review and meta-analysis of randomized controlled trials. Sci. Total. Environ..

[15] Rice MB (2013). Short-term exposure to air pollution and lung function in the framingham heart study. Am. J. Resp. Crit. Care Med..

[16] Langley SJ (2003). Exposure and sensitization to indoor allergens: Association with lung function, bronchial reactivity, and exhaled nitric oxide measures in asthma. J. Allergy Clin. Immunol..

[17] Gao YL, Tian EZ, Zhang YP, Mo JH (2022). Utilizing electrostatic effect in fibrous filters for efficient airborne particles removal: Principles, fabrication, and material properties. Appl. Mater. Today.

[18] Kelly FJ, Fussell JC (2019). Improving indoor air quality, health and performance within environments where people live, travel, learn and work. Atmos. Environ..

[19] Li CH, Bai L, He ZJ, Liu XR, Xu XL (2021). The effect of air purifiers on the reduction in indoor PM_2.5_ concentrations and population health improvement. Sust. Cities Soc..

[20] Zhang YP (2011). Can commonly-used fan-driven air cleaning technologies improve indoor air quality? A literature review. Atmos. Environ..

[21] Gao YL, Tian EZ, Mo JH (2023). Electrostatic Polydopamine-Interface-Mediated (e-PIM) filters with tuned surface topography and electrical properties for efficient particle capture and ozone removal. J. Hazard. Mater..

[22] Tian EZ, Gao YL, Mo JH (2023). Experimental studies on electrostatic-force strengthened particulate matter filtration for built environments: Progress and perspectives. Build. Environ..

[23] Zhang XY, Gao B, Creamer AE, Cao CC, Li YC (2017). Adsorption of VOCs onto engineered carbon materials: A review. J. Hazard. Mater..

[24] Chen HY, Mo JH, Xiao R, Tian EZ (2019). Gaseous formaldehyde removal: A laminated plate fabricated with activated carbon, polyimide, and copper foil with adjustable surface temperature and capable of *in situ* thermal regeneration. Indoor. Air..

[25] Chen QW (2023). Recent progress and perspectives of direct ink writing applications for mass transfer enhancement in gas-phase adsorption and catalysis. Small Methods.

[26] Huang CH (2021). Impacts of using auto-mode portable air cleaner on indoor PM_2.5_ levels: An intervention study. Build. Environ..

[27] Cooper E, Wang Y, Stamp S, Burman E, Mumovic D (2021). Use of portable air purifiers in homes: Operating behaviour, effect on indoor PM_2.5_ and perceived indoor air quality. Build. Environ..

[28] Pei JJ, Dong CB, Liu JJ (2019). Operating behavior and corresponding performance of portable air cleaners in residential buildings, China. Build. Environ..

[29] Malayeri M, Nasiri F, Haghighat F, Lee C (2023). Optimization of photocatalytic oxidation reactor for air purifier design: Application of artificial neural network and genetic algorithm. Chem. Eng. J..

[30] Wei JZ, Mo JH (2023). figshare.

[31] Cho, B. *et al*. Effective Missing Value Imputation Methods for Building Monitoring Data. *IEEE Int. Conf. Big Data*, 2866–2875 (2020).

[32] van Buuren S (2007). Multiple imputation of discrete and continuous data by fully conditional specification. Stat. Methods. Med. Res..

[33] van Buuren S, Groothuis-Oudshoorn K (2011). mice: Multivariate Imputation by Chained Equations in R. J. Stat. Softw..

[34] Shen C, Bao XJ, Tan JB, Liu ST, Liu ZJ (2017). Two noise-robust axial scanning multi-image phase retrieval algorithms based on Pauta criterion and smoothness constraint. Opt. Express.

[35] Jung W, Wang Z, Hong TZ, Jazizadeh F (2023). Smart thermostat data-driven U.S. residential occupancy schedules and development of a U.S. residential occupancy schedule simulator. Build. Environ..

